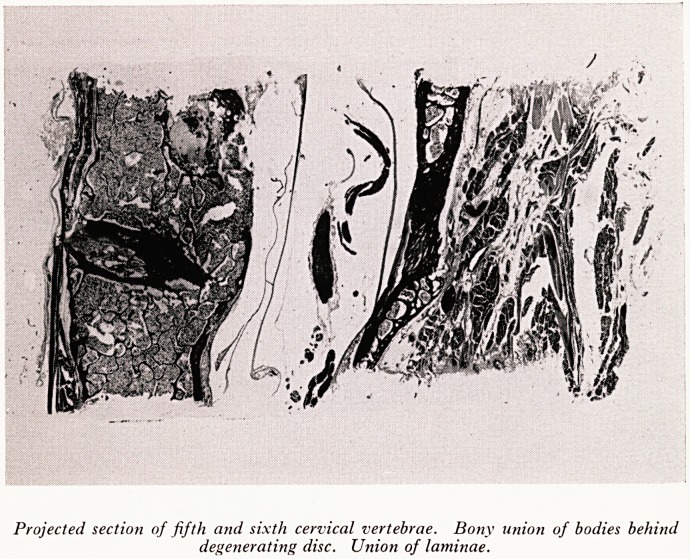# Ankylosing Spondylitis with Mitral Stenosis

**Published:** 1961-10

**Authors:** O. C. Lloyd


### ANKYLOSING SPONDYLITIS WITH MITRAL STENOSIS

*A Clinico-Pathological Conference of the University of Bristol held on the 24th January, 1961*

CHAIRMAN: DR. O. C. LLOYD

P.M. No. 6404

*Dr. Lloyd:* For the most part this man was under the care of Professor Perry and since he has most of the notes I have asked him to tell us the story.

*Professor Perry:* I first saw this man in 1945 when he was 35 with Dr. Bryan Adams at the General Hospital. He had been referred to Dr. Adams, with a view to having radiotherapy, by Mr. Pridie; and he had been referred to Mr. Pridie by Mr. Tasker who had initially seen him because of a hernia. I think this history may be significant because it obviously points to the fact that the man's back at that time was not giving him a lot of trouble, since he had consulted his doctor about his hernia. The story was that at the age of 28, in 1938, he began to get pain and stiffness in his back. He had a lot of pain in his back so that sometimes he had to sit up in bed at night to get relief from the pain, and he complained of a lot of morning stiffness. He declared that he had no other joint involved except his back and this trouble had increased gradually over the years. As I say it was not particularly worse in 1945 and he consulted his doctor because of the hernia. I forget whether Mr. Tasker operated on this occasion or not, but he was so horrified at his back that he sent him to Mr. Pridie who sent him to Dr. Adams. At this time his main complaint apart from his hernia was that the bones of his back were "locked", and he had difficulty in sleeping because he could not get comfortable in bed. This of course is very characteristic of the later active stages of ankylosing spondylitis. One of the most bitter complaints of these patients is that they cannot lie comfortably in bed. On examination he had severe deformity of his spine with a very marked kyphosis, and his spine was practically completely rigid from the lumbar region nearly up to his neck. I should have told you that the only thing in his history, previous to this illness in 1938, was that at school he was not allowed to swim because of a bad heart. When we examined him at the General Hospital we found no abnormality in his heart. His sedimentation rate was high. We came to the conclusion that he had ankylosing spondylitis, which was approaching the latent stage but that there was still some activity. We thought that it would be worth while treating him with X-rays and he received X-rays by what is commonly known as the Gilbert Scott technique. This is perhaps important because it is quite different from what is usually done nowadays. We saw him on and off through 1945. In 1946 the pain in his back was less and he thought he was keeping better. He now had pain in his neck. The radiotherapy he had had to date had not made very much difference. We thought we would see if we could help him with a brace. He wore the brace on and off, but said it didn't help him and it was more nuisance than it was good. The difficulty about a brace for this condition is that if it is to give the spine any support it has to be heavy.

In April 1946 he was complaining more of pain in his neck, however by August 1946 he was obviously better because he went back to work. He got a job as a long-distance lorry driver and didn't attend the hospital very regularly after this, I suspect because he didn't think he received much benefit and secondly because he was probably better. He carried on this job. When we did see him during 1946 his only complaint was that of neck pain.

In 1947 he had more radiotherapy and by March 1947 he had had eight courses by the Gilbert Scott technique. We came to the conclusion that it was questionable whether it did him any good. His sedimentation rate was still high.

In 1948 he was working very regularly and attended hospital very irregularly.

In 1949 he only had occasional pain, but his sedimentation rate was still high.

In 1950 he was still working and really had no complaints.

He was not seen from 1951 until 1957 when he was again referred to Mr. Pridie because the pain in his neck was severe and I think, although I cannot check this, he was given more radiotherapy. I did not see him at this time. However about the middle of 1954 he suddenly, over 3 days, developed intense dyspnoea and orthopnoea, and was admitted to Ham Green as an emergency. At Ham Green they found he had no fever, a regular pulse, and râles at both bases, particularly the left base, and he was diagnosed as having early acute left ventricular failure. Examination of his heart showed a loud apical systolic murmur. I should of course have said the deformity of his chest made it difficult clinically to tell whether or not his heart was enlarged, he was so bent. During his convalescence at Ham Green they also heard a diastolic murmur so the suggestion was that he had mitral stensis. X-rays taken at the time, about which I will tell you as I have them here, showed a bilateral pleural effusion, left more than right. Shadowing of both lower lobes suggested pulmonary oedema. His heart shadow was difficult to see because of the deformity of his chest but it looked as though both left and right ventricles were enlarged, the pulmonary artery was dilated and the peripheral pulmonary vessels congested. An X-ray taken later on, on his admission to Ham Green, showed clearing of the oedema and pleural effusion. His heart rhythm remained normal. He was discharged in August and in October was sent to me. He was still out of breath. As far as I could see his spondylitis was inactive. He had practically no pain but was rigid from top to bottom with a severe kyphosis and, of course, had very restricted chest movement and respiratory expansion. There was then no gross evidence of heart failure but we had this history of heart failure at Ham Green and the problem was why this man *had* heart failure. On examination at this time we found a tapping apex beat and, we thought, a pre-systolic gallop. His E.C.G. showed a slight right ventricular preponderance but nothing very striking. X-rays of his chest were difficult to interpret because of the deformity. Perhaps Dr. Evans will do it for you. The problem was why he had developed failure. Had he cor pulmonale from his chest deformity? At that time we had no details of his admission to Ham Green but obviously this would not fit because on his admission to Ham Green he had left ventricular failure which he would not have had with cor pulmonale although of course this is a hazard for people with this degree of chest deformity. Had he spondylitic heart disease? This is what really excited us because there is a rare and peculiar form of heart disease associated with ankylosing spondylitis that occurs in perhaps 5 per cent of cases. A characteristic feature of this is an aortic valve lesion. We could find no evidence of an aortic lesion in this man. Had he rheumatic heart disease? We could not at that time hear a definite pre-systolic murmur and he had gone into heart failure with a normal rhythm. This is not common with rheumatic heart disease. In November he had no symptoms of heart failure but now he came up complaining of pain in his buttocks and back and we thought that possibly he had a flare-up of spondylitis. In the meantime he had developed a hydrocele and just as before his main complaint had been of his hernia, now his main complaint was of the hydrocele. I was not very keen on having this chap operated on because he really was rather a physical wreck. We tried having his hydrocele tapped. But this got tedious; he had to have frequent tapping. Finally it was decided that we might operate on him. However before he came in to have this operation he was admitted in February 1958 again with intense dyspnoea. He had a systolic murmur and later on, during his convalescence, we found a diastolic murmur; this of course is a repetition of what happened at Ham Green. He was treated with digitalis and this seemed to relieve his dyspnoea so we were encouraged to think that this dyspnoea was due to heart failure. He had his hydrocele operated on uneventfully and went home.

In April and May 1958 his main complaint was of increasing pain and stiffness in his neck. During 1958 we took him in to hospital again because of his neck pain. He was given all sorts of analgesics, we tried him with aspirin, then phenylbutazone which is more successful in ankylosing spondylitis than in any other form of rheumatic disease; and because in 1958 there was much discussion about anti-malarials in "rheumatoid arthritis" he was tried on chloroquin. However, none of these did him any good. We again noted apical systolic and diasolic murmurs and the other thing we found then and did not explain was an eosinophilia of 13 per cent. In desperation we asked Dr. Tudway to give him more radiotherapy. This was given in August and September and there was probably some relief of his pain. In December 1958 he came up again, complaining of increasing dyspnoea and some pain in his chest. We thought that perhaps this was due to the fact that his digitalis had been reduced. We put him back on his digitalis without very much effect. X-rays showed pleural effusion, mostly on the left side, and what looked like some collapse in his left lower lobe. In January 1959 he came in as an emergency with severe chest pain, dyspnoea, fever, and cough with purulent sputum. The sputum yielded a growth of mixed organisms including *Haemophilus*. We thought possibly he had some kind of secondary pneumonia. He made a fair response to treatment with penicillin and streptomycin but during convalescence he developed the physical signs and X-ray evidence of pleural effusion. The X-ray again looked like a mixture of effusion and consolidation of his left lower lobe. Although we kept him in hospital a couple of months this showed no clearing and he was finally sent to a convalescent home. I saw him again towards the end of March, and he said he was better. X-rays still showed this collapse at the left base.

In May when he came up he said he was much better and wanted to try a light job, and we thought this was a good idea. But it did not last very long and next month his breathing was worse, and he now complained of pain going down his legs. This suggested that his spondylitis was perhaps flaring up again. This of course is what happens to patients with ankylosing spondylitis. Once you have got to the chronic phase you do have these flare-ups every now and again, in which the disease seems to become active and progressive. So, in desperation, we tried the only thing we had not tried, and that was steroids. He had fairly large doses of steroids without any effect except that in August 1959 he was admitted as an emergency with an acute gangrenous appendicitis. Despite the fact that he was then on steroids this was removed uneventfully. All this time his chest picture was unchanged radiologically but in October the radiologist said that he thought there was now some clearing of his left lower lobe. In December 1959 he was admitted with severe congestive heart failure. Apparently this had followed the development of auricular fibrillation, and now he presented much more the pattern of genuine rheumatic heart disease. He made a very poor response to treatment and shortly after he was admitted he suddenly developed a right-sided hemiplegia. This seemed to make the diagnosis quite clear as far as his heart was concerned. He had chronic rheumatic heart disease, mitral stenosis, auricular fibrillation, cerebral embolism. He deteriorated rapidly and died early in the new year. So finally we came to the conclusion that this long-standing case of ankylosing spondylitis had an associated rheumatic heart disease with mitral stenosis, auricular fibrillation, and a cerebral embolism.

*Dr. Lloyd:* Dr. Mobarek has come here to tell us about the radiotherapy.

*Dr. Mobarek:* This patient first came to the Radiotherapy Department in 1945, when he was 35 years old. During the next two years he was given about ten courses of X-rays. His first two treatments were according to the Gilbert Scott technique, in which the spine is irradiated in strips 25 cm wide, while the other parts are shielded. This technique is no longer used. After this he appears to have had little trouble and we lost sight of him until 1958, when on enquiry we found that he was still under Professor Perry's care. In that year he was sent to us again and we gave him a course of super-voltage irradiation to the cervical spine with the cobalt machine. This was because he was having pain in the neck. After treatment he thought that there had been a very definite though slight improvement.

*Professor Perry:* The form of treatment he had in 1958—was it the Gilbert Scott or was it the McWhirter technique? The point is that the Gilbert Scott technique makes use of lower penetration and lower voltage X-rays, which may be very important in view of the complications which have been described. The McWhirter technique which has entirely superseded the Gilbert Scott, uses high voltage X-rays, directed focally to the point which is believed to be at fault, i.e. the spine, and this was popularized towards the end of the war. As you know people with ankylosing spondylitis who have received X-ray treatment have a much higher chance of developing myeloid leukaemia than the general population during the following 10 to 20 years. This is very worrying. I believe that the incidence of leukaemia is related to the amount of radiation delivered to the bone marrow and I wonder whether the old-fashioned Gilbert Scott low-voltage X-ray "bath" might give a smaller dose to the marrow than the newer high-voltage therapy directed at part of the spine. Has Dr. Mobarek any comment on this?

*Dr. Mobarek:* There is not much difference. With low-voltage therapy (140 kV) the depth of penetration is relatively small, but there is differential absorption by the bone as compared with the soft tissues. In consequence a larger volume of the patient gets irradiated and this includes the ribs. With high-voltage therapy (250 kV) there is much more uniform radiation of bone and soft tissue, with deeper penetration and less scatter. As a result a smaller volume of the patient gets irradiated. The actual dose received by the bone is more easy to calculate, and in fact only 1200 R would be given in one course of treatment lasting 4 weeks. With the low-voltage method up to 2000 R were given. In this particular patient we tried to give 250 kV to the neck, but the pain was so severe that it was impossible to localize the high-voltage machine over his neck. It was for this reason that we put him under the cobalt machine.

*Professor Perry:* This was in 1958?

*Dr. Mobarek:* Yes, in 1958. As Professory Perry was saying, X-ray treatment is now only given to the areas affected by pain, such as is produced by muscle spasm.

*Question:* Is there any evidence that this relieves pain in ankylosing spondylitis?

*Dr. Mobarek:* Yes, there have been several reports between 1934 and 1954 here in England. Over 13,000 cases were treated by means of X-rays and over 60 per cent of them had some relief of pain. Sometimes relief of pain is due to the disease burning itself out, but it is claimed that X-ray treatment may bring this about more quickly.

*Professor Perry:* It is a very vexed matter, sir, and there is a difference of opinion. When you have only a 60 per cent success ratio it is obviously not very impressive. Dr. West analysed all our cases in Bristol and followed the natural history of the disease. He came to the conclusion that he had no evidence that radiotherapy had any effect whatever. Dr. Charles Ragan of the Presbyterian Hospital in New York, who has written probably one of the best reviews of this condition in *Medicine* in 1956, came to very much the same conclusion. This is a very difficult thing to assess, because you are dealing with a disease which arrests at any stage and then in this arrested chronic stage it flares up from time to time, but each relapse tends to be relatively short-lived. The disease goes through three phases which I think Dr. West perhaps first made clear. The first stage consists of recurring attacks of pain and stiffness, the second of continuous symptoms and constitutional upset, and the third of declining activity with minor relapses. Occasionally radiologists find typical X-ray changes in the sacro-iliac joints in patients with no symptoms.

*Question:* Professor Perry has told us of the changes of myeloid leukaemia following irradiation for ankylosing spondylitis; perhaps you or he can tell us of the changes of irradiation in these cases?

*Dr. Mobarek:* The report of the United Nations Scientific Committee on the effect of atomic radiation, published in 1958, states that between 1935 and 1954 there were 13,352 cases of ankylosing spondylitis treated by means of X-rays. Of these 28 died of leukaemia (mostly myeloid), an incidence of 2000 per million. This is to be compared with the over-all death rate per million from leukaemia in England and Wales, which in 1935 was 21, in 1945 was 34 and in 1954 was 49 per million. In the same series of 13,352 cases there were 12 deaths from aplastic anaemia.

*Dr. Lloyd:* Is there any danger of producing a radiation nephritis?

*Dr. Mobarek:* No. The areas treated are far away from the kidneys, and no cases of radiation nephritis have been reported in cases of ankylosing spondylitis.

*Dr. Lloyd:* Dr. Evans has come with some X-rays.

*Dr. Evans:* I would first like to discuss the problem of his ankylosing spondylitis. When Professor Perry first saw him his disease was at a later stage. Usually the sacro-iliac joints are the first to be affected in this disease. Initially slight irregularity of the margins of the joints is seen. Later bone sclerosis occurs around the joints and usually ankylosis ensues.

The radiograph of the pelvis taken in 1947 shows complete fusion of the sacro-iliac joints. New bone formation is seen arising from the ischial tuberosities and also along the iliac crests at the site of tendon attachments.

The dorsal and lumbar vertebral bodies have a straight anterior margin; normally they are slightly concave. The anterior margins of the vertebral bodies are joined together by a bridge of calcification. The apophyseal joints are ankylosed and there is a marked kyphosis affecting the cervico-dorsal region of the spine.

A radiograph of the chest taken in January 1959 shows the heart is enlarged and the pulmonary arteries prominent. A diffuse opacity is present in the left lower lobe, the appearances being consistent with pneumonic consolidation. The pneumonia was slow to clear.

A further chest radiograph in December 1959 showed that the left lung was clear but there was an opacity at the right base limited by the oblique fissure. This is again consistent with pneumonic consolidation.

*Professor Perry:* May I ask Dr. Evans to tell us what he thought about these X-rays from Ham Green 18 months before that.

*Dr. Evans:* The heart is enlarged in its transverse diameter and the pulmonary arteries are also larger than normal. Diffuse opacities extend outwards into the lung fields from the hilar regions. There is probably a small left basal effusion. These appearances are consistent with left ventricular failure. The second radiograph taken two weeks later shows that the pulmonary oedema has resolved.

*Dr. Lloyd:* This man was very much wasted, but that was not the most remarkable thing. He had such a severe degree of kyphosis that lying as he was in the supine position the occiput was 27 cm off the table. The degree of angular curvature of the spine you can see from this median saggital slice. The cut is not quite straight in the middle, but then his spine was not quite straight; there is a distinct scoliotic curve in addition to the kyphotic curve which you see.

When the disease, beginning in the lower part of the spine, results in this degree of kyphosis, then compensatory extension takes place in the cervical part. Later that, too, became ankylosed, and by the time he came to me the whole spine was fixed, except for the atlanto-occipital joint.

There were some old adhesions to the appendicectomy scar in the abdomen, otherwise that was all right. Pleural cavities: there was almost complete obliteration of the cavities by fibrous adhesions, those at the left lower lobe being the toughest. I think that was where he had most of his pneumonic trouble. The pericardium was natural.

The heart weighed 360 g, a little on the heavy side, and he had moderate dilatation of all the chambers, but particularly of the left auricle. There was only a slight degree of hypertrophy of the myocardium. He had a little healed infarct at the apex of the left ventricle. Now the changes in the valves: the mitral and aortic valves were particularly involved, the condition being indistinguishable from healed rheumatism.

In the mitral valve (Plate XXIV) the aortic cusp was greatly thickened. The commissures were both closed, one of them was not only surrounded by fibrous tissue but also ulcerated and thrombus was forming upon it. This thrombus had broken off leaving a raw area and had produced embolism to the brain. It also produced embolism to the kidneys and there were two or three fairly recent infarcts to be found in those organs. There was also some thickening of the chordae tendineae, very much as you get in rheumatic heart disease which has burnt itself out.

The aortic valve cusps were a little thickened and one of the commissures was united completely and had already undergone calcification. The aortic ring itself was normal and the aorta showed only a moderate degree of atheroma. I was unable to find any of the changes in the aorta which are supposed to be characteristic of some cases of spondylitis. Moreover I was not able to find any changes which could be attributed to the X-ray therapy. The only changes which I did find in the way of abnormalities were down in the abdominal part of the aorta where there was a fair amount of atheroma and where there was some medial thinning and loss of elastic to go with it, but no more than you often find in cases of uncomplicated atheroma.

A projected section of the mitral valve is shown (Plate XXV). The dark line down the centre of the cusp represented the original thickness of this cusp. Additional and fibrous tissue had subsequently been plastered on to the surfaces both of the cusps chordae tendineae, during the slow process of this disease, which resulted in thickening of the valve. It was more conspicuous on the contact margin where it was laminated, as though layer upon layer of fibrin had been laid down and subsequently converted into fibrous tissue. The distal part of the original cusp was a bit frayed and vascular, but nowhere was there any severe scarring and distortion of the pattern, as is sometimes seen after active rheumatism. There were no Aschoff nodules in the myocardium or anything I could identify as a healed rheumatic lesion. In sum, the changes were those to be found in some cases of healed rheumatism, but did not contain any of the specific rheumatic hall-marks.

The left middle cerebral artery (Plate XXVI) was completely obstructed by an embolus, which probably originated from the mural thrombus on the mitral valve. At any rate the pattern of the occluding thrombus was not such as is seen arising in an artery, and moreover the walls of the artery, though greatly distended, were normal at this point. On the contrary the embolus showed on section columns of platelets characteristic of mural thrombi, such as form inside the heart. The obstruction caused infarction of the greater part of the left cerebral hemisphere.

In his gastro-intestinal tract I found that he had a healed gastric ulcer and also a healed duodenal ulcer.

The lungs were not particularly on the heavy side, but the right was heavier than the left. There was well marked collapse, particularly of the left lower lobe and early bronchopneumonia but the lungs were solid to the feel, apparently due to an old organized pneumonia and sections show that this was probably the case. They also show that the arteries and arterioles had undergone the kind of sclerotic changes one associates with mitral stenosis. The bone marrow was natural in the spine but I did not see it in the femora. I do not think that the X-rays had caused any damage to the bone marrow at all. The kidneys I have mentioned. The right testis was missing because they had removed it in treating his hydrocele. The adrenals showed abundant lipid in cortices of normal width. I am not quite sure how long ago it was you stopped giving him cortisone but anyhow, if it had produced any changes in the adrenal cortex, it had gone back to normal again; but I did find a cortical adenoma (1·2 cm in diameter) in the right adrenal.

Now with regard to the bones, most of the spine was removed for later examination and just in case anyone asks me what the sacro-iliac joints looked like I have to confess I left them behind in the body for two reasons: firstly because I was particularly keen on finding what the *early* changes were in this disease and the sacro-iliac joint is one of the first to get involved and I reckoned that probably I should find changes there, if at all, of an even later type than those which I was going to find elsewhere in the spine; and the other reason is that when one removes a large portion of spine for subsequent examination one has to use a prosthesis in the form of a broom stick. We keep quite a number of broom sticks in the post-mortem room for this purpose and one has to have something at each end to fit it into, the skull at one end and the sacrum at the other. After fixing the spine I cut the median sagittal slice which I have shown you and another slice next to it (parasagittal) for histology. I had both of them X-rayed, which not only showed beautifully the minute changes of bone structure but also was a guide as to which pieces to take for histology.

An X-ray of the median sagittal slice of the spine (Plate XXVII) shows that the sclerosis and union of the anterior parts of the lower thoracic and lumbar vertebral bodies was not due, in this case, to ossification of the anterior spinal ligament. It was due to condensation in the cortex and ossification of the anterior parts of the intervertebral discs, so that the vertebral lips had grown towards one another. Union has occurred particularly in the concavities: anteriorly in the lower spine, posteriorly in the cervical region. The arches, laminae and their articulations were fused to form a more or less continuous process all the way down. There has been some calcification in the centres of some of the cervical intervertebral discs. That, I gather, is an uncommon but quite well recognized complication of ankylosing spondylitis.

An enlargement of the lower thoracic part of this X-ray (Plate XXVIII) shows the details even more clearly. In the laminae the pattern of the bone has, as it were, woven itself into a continuous whole with loss of the articular joints. The union of the anterior parts of the vertebral bodies is well shown. The union is often preceded by a certain amount of calcification of the cartilage, particularly at the margins, where union is more likely to occur.

In X-rays of slices of the sterno-clavicular joints (Plate XXIX) the very early changes of this disease are well shown. In the left there is still fairly good cartilage on the clavicle, but that on the sternum has undergone a considerable degree of erosion. In the right joint the process was even more marked. In both the sclerosis of the bone near the joint is well shown. At autopsy the right joint was very much redder and more vascular looking than the left. These changes, I believe, are only distinguishable from those of rheumatoid arthritis by the bony sclerosis around and inside the joint. Inboth conditions there is the same destruction of cartilage and joint space with proliferation of the synovial membrane.

Projected sections of the left (Plate XXX) and right (Plate XXXI) sterno-clavicular joints illustrated these points. On the left the sternal cartilage showed fibrillary disintegration and erosion. At one particular point can be seen a pannus of synovium growing out of a crack in the cartilage. There is an increase in the amount of synovial tissue and everywhere can be seen in and around the joint densification of the bone. Round cell infiltration of the inflamed synovium, so often seen both in rheumatoid arthritis and in ankylosing spondylitis, is absent. On the right the cartilage had more completely disintegrated and given way to bony overgrowth and to vascular granulation tissue.

A later stage of the process was shown in a projected section of the apophysial articulation between the 3rd and 4th cervical vertebrae (Plate XXXII). These are the joints which are supposed to get fused first in the spine. Their fusion precedes that of the intervertebral discs. The section shows the remains of two layers of cartilage. The joint space between them has disappeared and they are united by a thin layer of

PLATE XXIV

*Mitral valve. Fibrous thickening of cusps and of chordae tendineae. Union of commissure and ulceration with mural thrombosis. Emboli started at this site.*

PLATE XXV

*Mitral valve, projected section (Phloxine-tartrazine). The thin dark line represents the original valve cusp. There is some fraying and vascularization of this near its free margin. Parallel bands of collagen laid down on its surfaces and on the chordae tendineae cause thickening.*

PLATE XXVI

*Base of brain. Embolism of left middle cerebral artery.*

PLATE XXVII

*X-rays of thin median sagittal slice of spine and of thin slices of sterno-clavicular joints.*

PLATE XXVIII

*X-ray enlargement of sagittal slice of spine in lower dorsal region. The slice is off-centre and shows union of the laminae and apophysial joints.*

PLATE XXIX

*X-ray of slices of sterno-clavicular joints showing early destruction and sclerosis.*

PLATE XXX

*Projected section of left sterno-clavicular joint. Early destruction of cartilage with synovial pannus. Bony sclerosis on each side of joint with lipping.*

PLATE XXXI

*Projected section of right sterno-clavicular joint. Almost complete destruction of carti-large, synovial proliferation and bony sclerosis.*

PLATE XXXII

*Projected section showing remains of apophyseal joint between third and fourth cervical vertebræ. Bony union on each side.*

PLATE XXXIII

*Projected section of fifth thoracic vertebra. Early changes in front of disc. Obliterated apophyseal joints.*

PLATE XXXIV

*Projected section of fifth and sixth cervical vertebrae. Bony union of bodies behind degenerating disc. Union of laminae.*

fibrous tissue. Around both sides of the joint there is bony union. The pattern of this bone is that of medullary bone, normal except for the greater thickness and number of its trabeculae. Additional bone can be seen to have been laid down outside the original cortex. The longitudinal spinal ligaments were not found to have been ossified.

The sclerosis around the intervertebral discs is shown in Plates XXXIII and XXXIV. Plate XXXIII is a projected section of the 4th thoracic vertebra, showing a fairly early lesion. There has been already some collapse of the intervertebral discs, though that is not the first thing that happens as a rule. Here it is becoming invaded by bone from the margins of the vertebral bodies above and below.

Plate XXXIV, a projected section of the 5th and 6th cervical vertebrae shows a later stage of ankylosis. There is complete bony union across the gap, where in the previous section it was only beginning. There is some calcification in the nucleus pulposus.

A projected section of two lumbar vertebrae showed complete fusion of the laminae.

That completes the description of this case and I will leave it to other people to unravel the interesting question of the relationship of this disease to rheumatoid arthritis, and of the associated heart disease to either.

*Professor Perry:* The whole problem of ankylosing spondylitis is first of all bedevilled by terminology and there is an acute cleavage on the two sides of the Atlantic as to what to call it and what it is. Nearly all the Americans call it rheumatoid spondylitis and say it is merely a variant of rheumatoid arthritis. We say that whatever it is, it is *not* rheumatoid arthritis. That is a gross generalization but that is what it comes to.

I think anyone who analyses clinically a large number of cases of ankylosing spondylitis finds that about four-fifths of them fall into what might be called the typical pattern and there is about one-fifth where there is something atypical. The things that are atypical are first of all, not in any special order, there may be an associated psoriasis. This of course puts us back into the whole question of the relationship with rheumatoid arthritis because as you know you do see "rheumatoid arthritis" associated with psoriasis. Some people say it is different and call it psoriatic arthritis and so on. Then there is another group of patients whose "ankylosing spondylitis" starts fairly definitely with an attack of what we call "Reiter's syndrome". The vast majority of people I think who have Reiter's syndrome ultimately recover with very little permanent joint disability. But there is a group who gradually finish up with a clinical picture of ankylosing spondylitis. Then there is another group who start with involvement of peripheral joints as though they were going to have rheumatoid arthritis. This morning I saw a girl with just this story. She had been treated for several years as rheumatoid arthritis and now shows a clinical picture of ankylosing spondylitis. The peripheral joints show very little wrong. Perhaps it is only fair to say that Dr. Middlemiss says that her X-rays show a very atypical ankylosing spondylitis. I think her heart is normal. Then there is another group of patients who develop a picture of ankylosing spondylitis except that during the early part of their illness they have recurrent attacks of acute peripheral polyarthritis which is preceded by an upper respiratory infection and which clinically is indistinguishable from acute rheumatism, and it is these people very often who develop rheumatic heart disease. For instance in the series studied by Ragan and Broomberg 25 out of 128 patients had some heart symptom or signs. Of those with evidence of valvular disease of the heart 70 per cent gave a history of what appeared to be attacks of rheumatic fever. Of those who had no evidence of valvular heart disease 8 per cent gave such a history. Now this man had no such history at all, and yet he had a heart lesion which Dr. Lloyd says is typical of rheumatic heart disease. Is this a chance association of rheumatic heart disease and ankylosing spondylitis? After all, rheumatic heart disease is common enough, and it must occasionally happen that the same man gets both. Is this spondylitic heart disease? I have only seen three specimens of this, two were in Boston and the slide of the third I

have is by courtesy of Dr. Crowe. The damage is at the root of the aorta. The first one I saw I thought was a peculiar syphilitic aortitis and then when I looked more closely I saw that it was something quite different.

Dr. Lloyd was very careful to point out to us that the root of the aorta was normal whereas it was the valve cusps that were a little puckered. The other thing is that these people who have recurrent attacks of "rheumatic fever" at the onset of ankylosing spondylitis do get permanent changes in their peripheral joints. They develop what is sometimes called the Jaccoud arthritis, which I must confess I have never seen. This is said to be a very rare complication of rheumatism. This man again did not have that, in fact this man clinically apart from his sterno-clavicular joint had no involvement of peripheral joints. Typical ankylosing spondylitis does spread to the peripheral joints, as a rule involving the larger proximal joints and only very late, if at all, it involves the small peripheral joints that are commonly involved in classical rheumatoid arthritis.

*Dr. Gibson* (in answer to a question): The antistrepsolysin-O test is very valuable in the ordinary acute rheumatic cases but in the type of heart disease associated with ankylosing spondylitis it probably would be negative.

*Dr. Lloyd:* Dr. Gibson, would you like to come and talk about that?

*Dr. Gibson:* This case shows, in my opinion, an association of conditions. It is probably ankylosing spondylitis, plus a very slow-going atypical acute rheumatic heart lesion. The history in youth was that he had a damaged heart. He probably had clinical juvenile rheumatism and at that time this process started to develop. Ankylosing spondylitics not infrequently combine their main disease with others. Dr. Middlemiss published a very interesting one of ankylosing spondylitis with rheumatoid arthritis, the two diseases running their perfectly characteristic courses. Dr. Hill had a case within the last few months of ankylosing spondylitis with some very unusual features. We thought of L.E. but no L.E. cells were found. It went up to one of the London hospitals and they found aminoaciduria and idiopathic hypocalcaemia with other biochemical abnormalities of a genetic type, which reminds us that ankylosing spondylitis is essentially genetic and probably you can have combined or linked defects. We wonder whether in this case there was any family history just to help clinch the diagnosis. I have not seen a case of ankylosing spondylitic aortitis and I think it must be rather rare. Our trouble is that people don't die now in hospital with ankylosing spondylitis. When I came to Bath in 1935 there were any number of them as almost permanent inmates in the rheumatic hospitals, going round bent like question marks. Now that the orthopaedic surgeons' activities are bearing fruit these patients maintain a good posture, with a good vital capacity. Deep X-rays may or may not help, but whatever the cause, most spondylitics are now doing a full-time job. We only see them now in Outpatients coming for a check-up. It is a long time since we have had a post mortem on one. I think on the whole I would say this is a combined, linked condition of two normally unrelated rheumatic diseases.

*Professor Perry:* Dr. Gibson asked about a family history, sir. I can say with complete confidence that there was no family history in this man's case. This was one of the cases Dr. West particularly studied from the point of view of family history when he wrote his paper showing that there was a family history in many of these cases.

I wonder, sir, if I might ask Dr. Evans some questions because this story of recurrent attacks of polyarthritis in people with ankylosing spondylitis comes from the Manchester school and it seems to me that it is only seen in Manchester, but there have been two or three reports in the *Annals of Rheumatic Diseases* and in the *B.M.J.* from Thomas and from Sharp in Manchester and they say that the radiological picture does show differences in the patients starting with "acute rheumatism" from that found in the classical type. These differences which they have described are that the sacro-iliac joints do not show absolute fusion, but spotty areas of sclerosis and areas of erosion, that the interfacetal joints show fusion, that there is narrowing of the intervertebral discs with ossification.

One other thing: it is said that whether or not X-ray therapy helps the 80 per cent of typical cases it is no good whatever in the atypical cases and it seems to me, reviewing this man, that although he had a great deal of radiotherapy he really was not very much better for it.

*Dr. Evans:* I agree that there are reports on the radiographic differences between ankylosing spondylitis and the changes in the spine associated with rheumatoid arthritis. It would seem that the essential difference lies in the severity of the disease in the sacro-iliac joints. Sometimes in rheumatoid patients and also in those with Reiter's syndrome one sacro-iliac joint may be more severely affected than the other. On the other hand if ankylosis occurs on both sides it is likely to be a true case of ankylosing spondylitis. The increased height of the vertebral bodies in ankylosing spondylitis is I think an established fact.

*Mr. Pridie:* Whereas the clear-cut case of ankylosing spondylitis can be diagnosed by anybody at a glance, the early case is extremely difficult.

I always remember a very advanced case we had at Winford. The patient was so flexed that his hair hung vertically from his head and he walked backwards looking through his legs. I had taken him in to perform an osteotomy of his spine but the operation never came off. A similar case was operated on by another surgeon the previous day, and, unfortunately the patient died in the night. My patient thereupon fled in the morning and he has never been seen at the hospital since. It is quite a dangerous procedure, of course, to straighten up a patient who is so deformed, but it is quite possible in a severe deformity to perform an osteotomy of the spine and leave the patient in a vertical position, which greatly aids his comfort and well-being.

The results of X-ray treatment, on the whole, have not impressed me. In certain cases the disease makes a natural halt and does not progress. The worst cases, of course, tend to come to X-ray for treatment because those treating them are desperate, but I am sure that there are many minor cases which never have X-ray treatment and the disease does not progress. I know of one doctor doing active work in Bristol who possesses fused sacro-iliac joints and the disease has never progressed in his case. He leads an active life, plays games, and it does not worry him in the slightest.

The diagnosis of the early cases is difficult but it is most important that it should be made. Patients with this disease must be kept mobile. The first physical sign which leads the doctor to a diagnosis of ankylosing spondylitis is diminution of rotation of the lumbar spine. This can be noticed long before there are any X-ray changes. The next most important aid to diagnosis is diminution of chest expansion. A very diminished chest expansion in otherwise healthy young men—and these cases are often very robust and athletic types—is ominous. The first thing one notices on examining them is that they only have a chest expansion of one inch or less and you can be quitecertain that such a patient has an early ankylosing spondylitis.

A lot can be done by a course of breathing exercises and they can regain their normal chest expansion and prevent the joints ankylosing.

To diagnose ankylosing spondylitis cases have to be watched carefully and, if it progresses, there will eventually be sclerosis of the sacro-iliac joints and limitation of rotation of spine. Pain may be first experienced in the sternal joints. All these factors will lead one to the diagnosis. Very often the sedimentation rate is quite normal.

There are a host of patients with early ankylosing spondylitis who live useful lives and hold down a job without undue trouble. In these cases it is most important to keep them active. They must not be put to bed or treated with a plaster cast. They must be actively kept on their feet. A really severe case progresses like a forest fire and very little can be done for the patient, who can end up with very severe ankylosis affecting all the major joints.

For treatment Delta Butazolidin is very useful and seems to give them more relief than almost any other drug.

*Dr. Lloyd:* The reason, which I do not think has been mentioned so far, for the diminution of chest expansion is because one of the earliest joints in the spine or by the spine to get affected is the articular joint at the posterior end of the rib with the vertebra. It gets affected almost as soon as the intervertebral articulations, the apophyseal joints.

I think we should be very grateful indeed to all the people who have come here today to tell us about this case; it has been particularly fascinating and I hope you have enjoyed hearing about it.

## Figures and Tables

**Figure f1:**
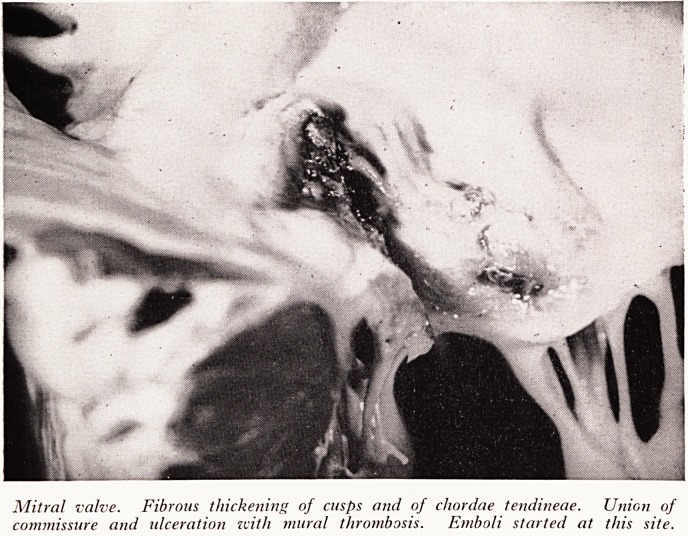


**PLATE XXV f2:**
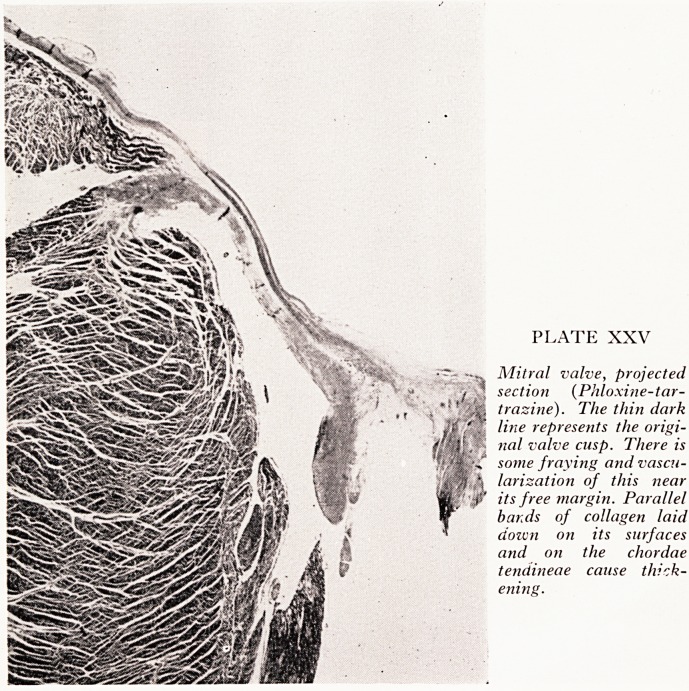


**Figure f3:**
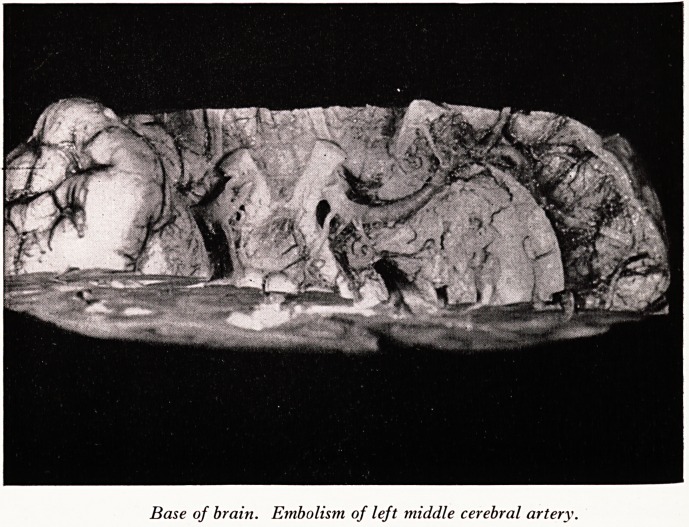


**PLATE XXVII f4:**
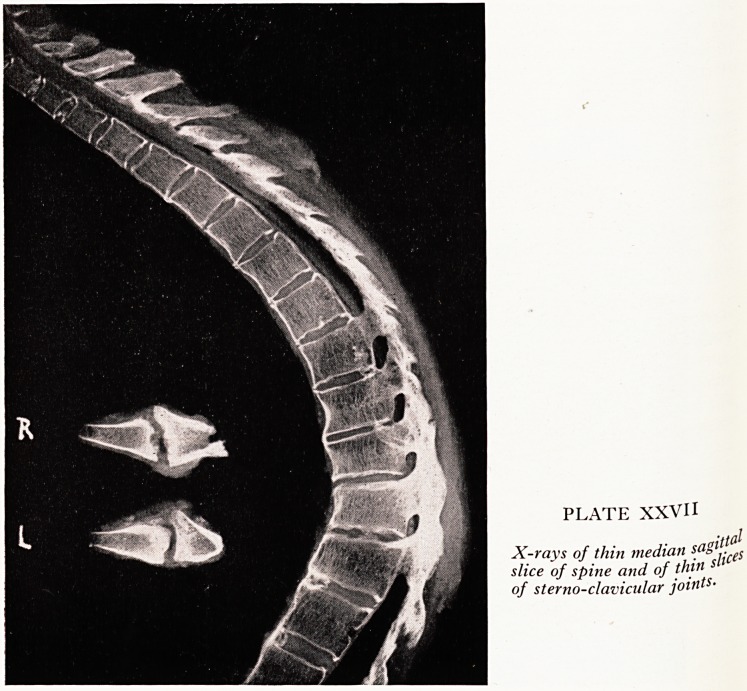


**PLATE XXVIII f5:**
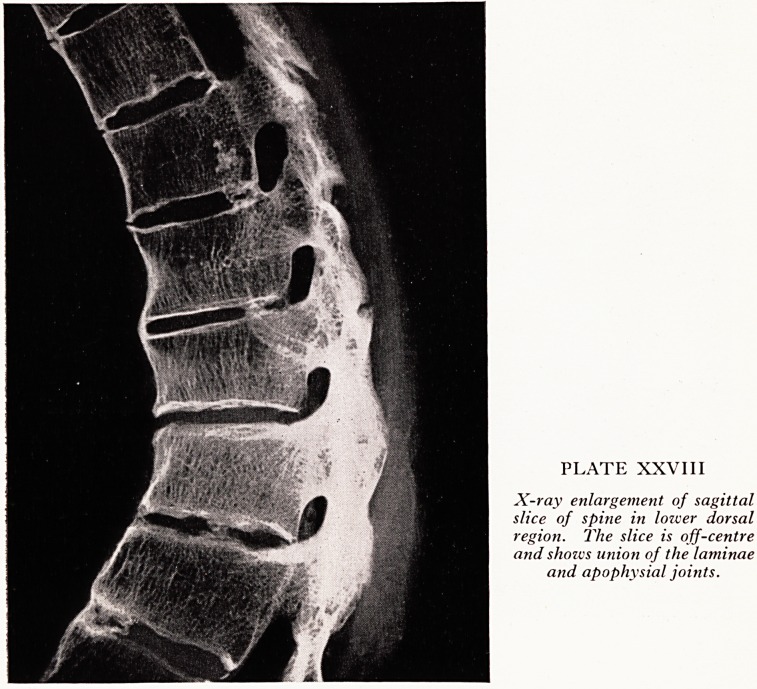


**PLATE XXIX f6:**
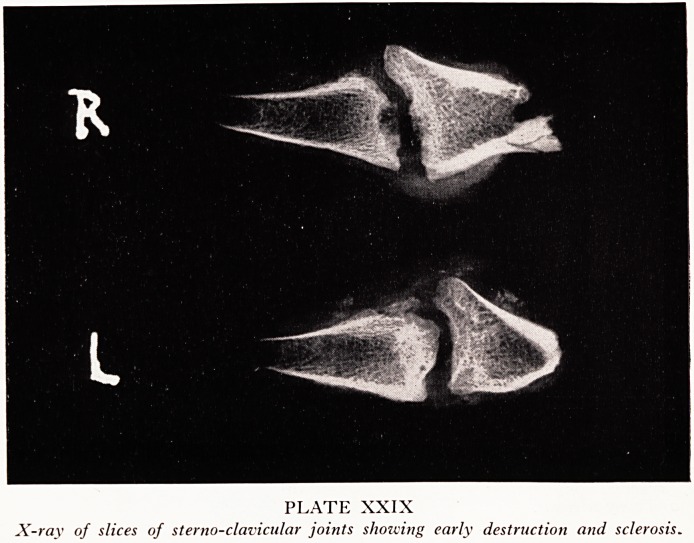


**PLATE XXX f7:**
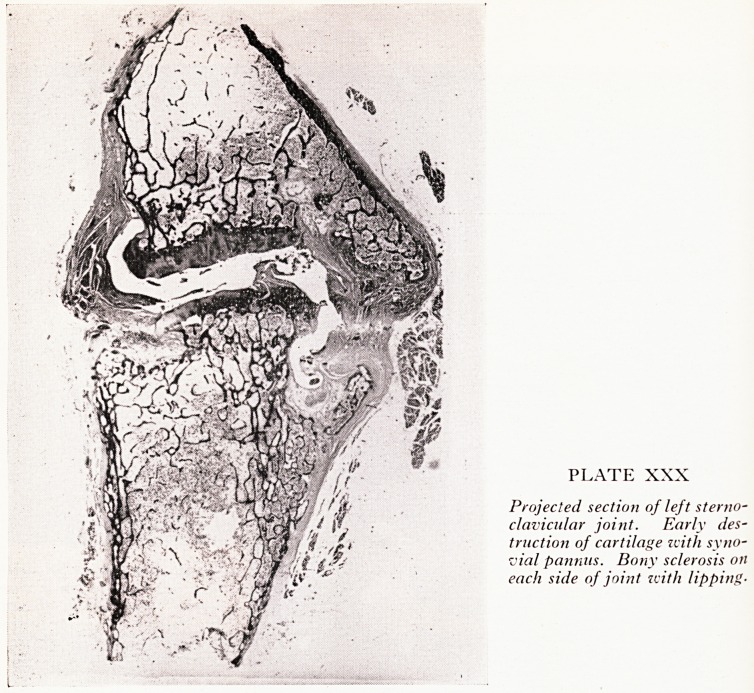


**PLATE XXXI f8:**
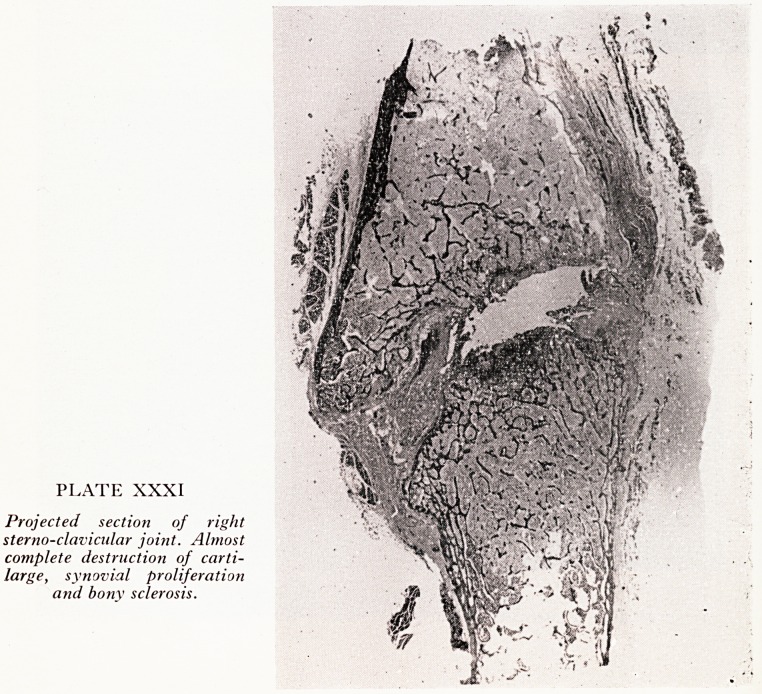


**Figure f9:**
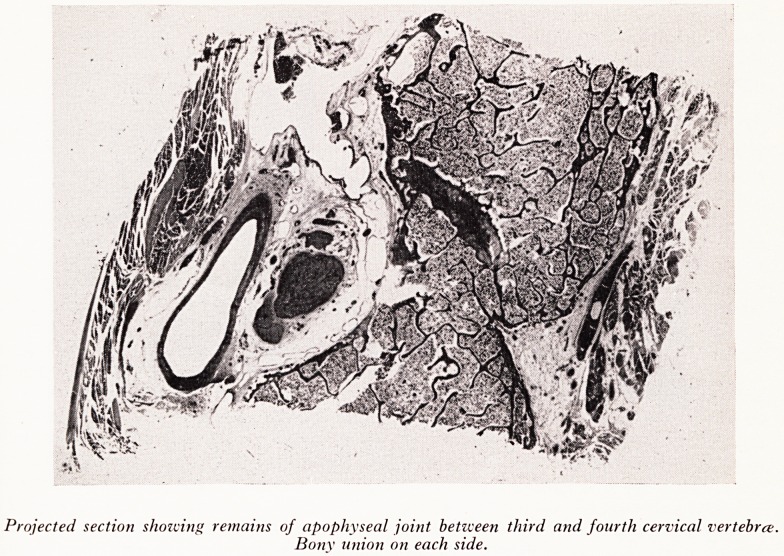


**Figure f10:**
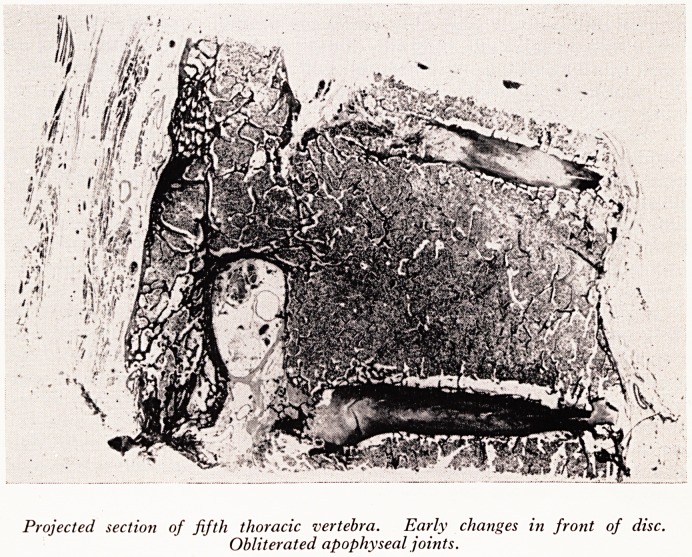


**Figure f11:**